# Managing Primary Bladder Neck Obstruction in Females: Is Bladder Neck Incision the Best Way Forward?

**DOI:** 10.7759/cureus.82976

**Published:** 2025-04-25

**Authors:** Skandh Bhatia, Narendra Kurmi, Sameer Vyas, Saurabh Jain, Amit Jain, Saurabh Kumar, Ajaybhai Jani, Gursharan Singh Mehta

**Affiliations:** 1 Department of Urology, Gandhi Medical College, Bhopal, Bhopal, IND; 2 Department of Community Medicine, Gandhi Medical College, Bhopal, Bhopal, IND

**Keywords:** bladder neck incision, bladder neck incision technique, bladder outlet obstruction, complications of bladder neck incision, lower urinary tract symptoms (luts), primary bladder neck obstruction

## Abstract

Background

Primary bladder neck obstruction (PBNO) in females is a rare condition with a tedious diagnostic process. The existing literature on bladder neck incision (BNI) to treat PBNO lacks a precise description of the surgical technique.

Objective

In this study, we provide a diagnostic protocol to streamline the evaluation of PBNO in females. We evaluate the outcomes of a precise surgical technique of BNI aimed at reducing the rates of known complications such as vesicovaginal fistula (VVF) and urinary incontinence.

Methods

This single-center prospective observational study, conducted over five years (2019-2024), included 20 patients diagnosed with PBNO who underwent BNI. Analyses included patient demographics, clinical presentation, treatment outcomes, and complications. Statistical analysis was performed using ANOVA, and univariate analysis was also conducted. Differences with P < 0.05 were considered statistically significant.

Results

At six months post-surgery, the mean maximum urinary flow rate (Qmax) increased from 6.49 ± 2.63 mL/sec to 12.41 ± 2.42 mL/sec (P = 0.0421). The mean post-void residual volume (PVR) decreased from 202.11 ± 70.20 mL to 53.11 ± 14.78 mL (P = 0.0152). The mean International Prostate Symptom Score (IPSS) decreased from 26.95 ± 2.84 to 14.74 ± 3.23 (P = 0.0325). The mean quality of life (QoL) score improved from 4.70 ± 0.80 to 1.60 ± 0.12 (P = 0.0067). None of the patients developed VVF or urinary incontinence in the post-operative period. One patient (5%) required re-surgery due to recurrence of bladder neck obstruction.

Conclusion

BNI for PBNO provides satisfactory results. A precise operative technique, with careful consideration of the depth and distal extent of the incision, helps avoid complications such as VVF and urinary incontinence.

## Introduction

Bladder outlet obstruction (BOO) in the female population presents as lower urinary tract symptoms (LUTS) [1]. Compared to men, BOO in women is not a common condition and is found in about 2.7-8% of women who are evaluated for LUTS [2,3]. The etiological causes of BOO in women can be functional or anatomical obstruction [4]. The more common of these are anatomical and can be divided into extrinsic (pelvic organ prolapse, uterine tumor, or post-anti-incontinence procedure), urethral (stricture, meatal stenosis, urethral caruncle, or diverticulum), or luminal (stone, bladder or urethral tumor, or foreign body). Functional causes may be due to impaired detrusor contractility or improper external sphincter relaxation or contraction during voiding caused by neurological diseases, senile bladder changes, diabetes mellitus, or non-neurogenic causes. The two most common non-neurogenic functional causes are dysfunctional voiding (DV) and primary bladder neck obstruction (PBNO) [5,6]. PBNO presents with storage or voiding-type LUTS, or with urinary retention as the first symptom [7].

The existing studies that evaluate bladder neck incision (BNI) as a treatment for PBNO establish BNI as an effective treatment. However, there are some procedure-associated complications (recurrent bladder neck obstruction, vesicovaginal fistula (VVF), urinary incontinence). In this study, we describe a precise surgical technique of BNI with considerations to avoid or minimize the aforementioned complications.

## Materials and methods

This prospective observational study was undertaken at the Department of Urology, Gandhi Medical College, Bhopal, India, from January 2019 to January 2024.

Inclusion criteria

Females diagnosed with PBNO (using our institutional diagnostic algorithm) and managed by the precise surgical technique of BNI were included in this study.

Exclusion criteria

All cases of BOO where the etiology was determined to be anatomical or neurological in nature were excluded.

Sample size calculation

PBNO in females is a rare diagnosis, and there is no available literature on its prevalence in an Indian population subset [2,3]. Therefore, all cases of PBNO diagnosed in the out-patient department at our center during the study period were included. A total of twenty cases were recruited during the study period.

**Figure 1:**
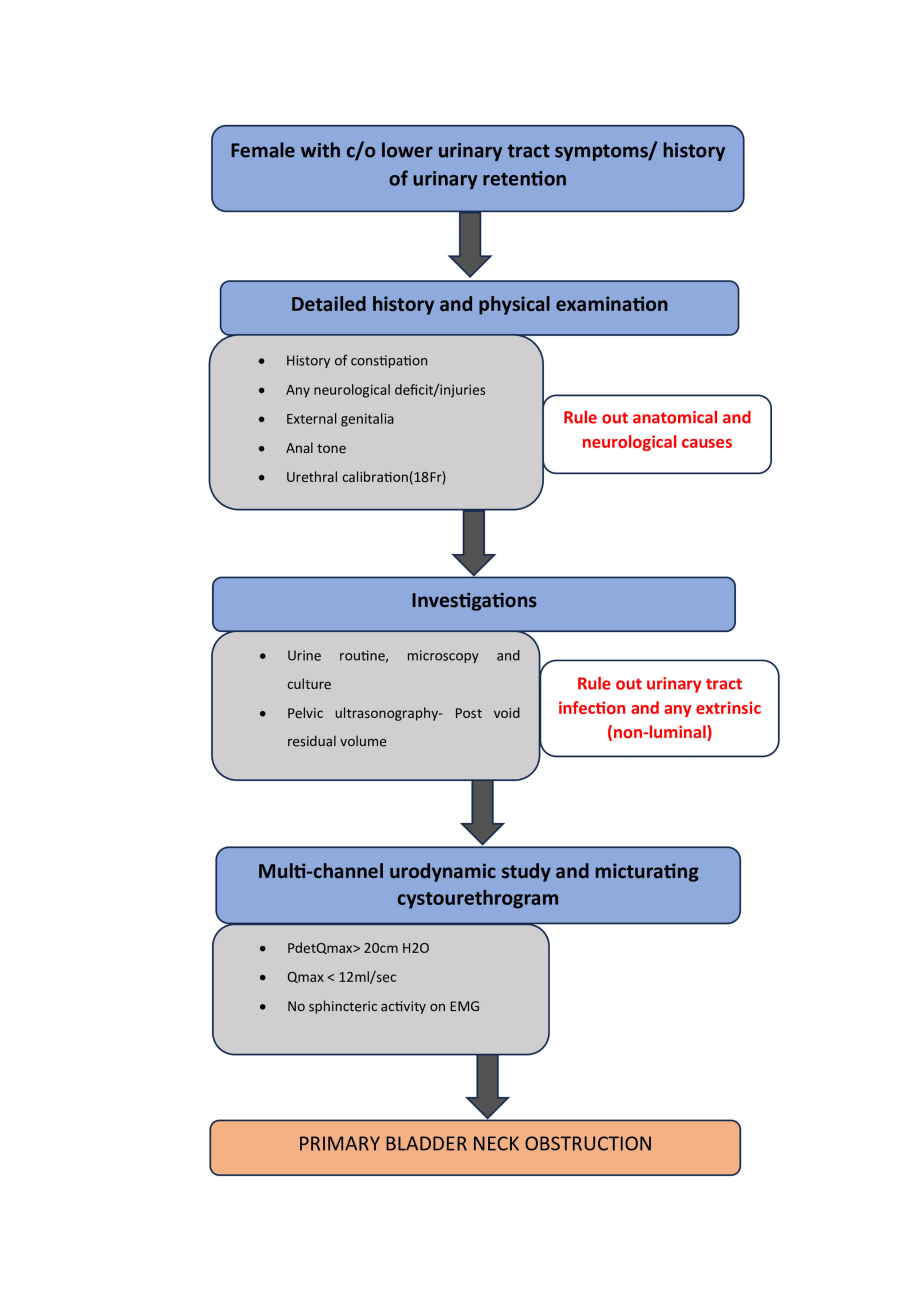
Institutional diagnostic algorithm to diagnose PBNO in females. Following this algorithm streamlines the diagnostic process, rules out differential diagnoses, and avoids over-investigation. PBNO: Primary Bladder Neck Obstruction.

The study commenced after obtaining ethical clearance from the Institutional Ethics Committee of Gandhi Medical College and Hamidia Hospital, Bhopal. Written informed consent was obtained from all participants, and their identities were kept confidential. The study adhered to the Declaration of Helsinki. We developed and used our institutional diagnostic algorithm (Figure 1) to diagnose PBNO in females. A comprehensive history was taken, and a detailed physical examination was performed. Urine routine and culture were analyzed to rule out urinary tract infection. Pelvic sonography was performed to measure post-void residual urine (PVR). In patients whose calibration with an 18 Fr Foley catheter was successful, urethro-cystoscopy was performed using a 19 Fr rigid cystoscope, and findings were recorded. Uroflowmetry was done to note the maximum urinary flow rate. All patients underwent urodynamic study and micturating cystourethrogram (MCU). The urodynamic diagnostic criteria for PBNO were a maximum flow rate (Qmax) < 12 mL/s, detrusor pressure during Qmax (PdetQmax) > 20 cm H₂O, with a silent sphincter on electromyography (EMG) [8,9].

Operative technique

**Figure 2:**
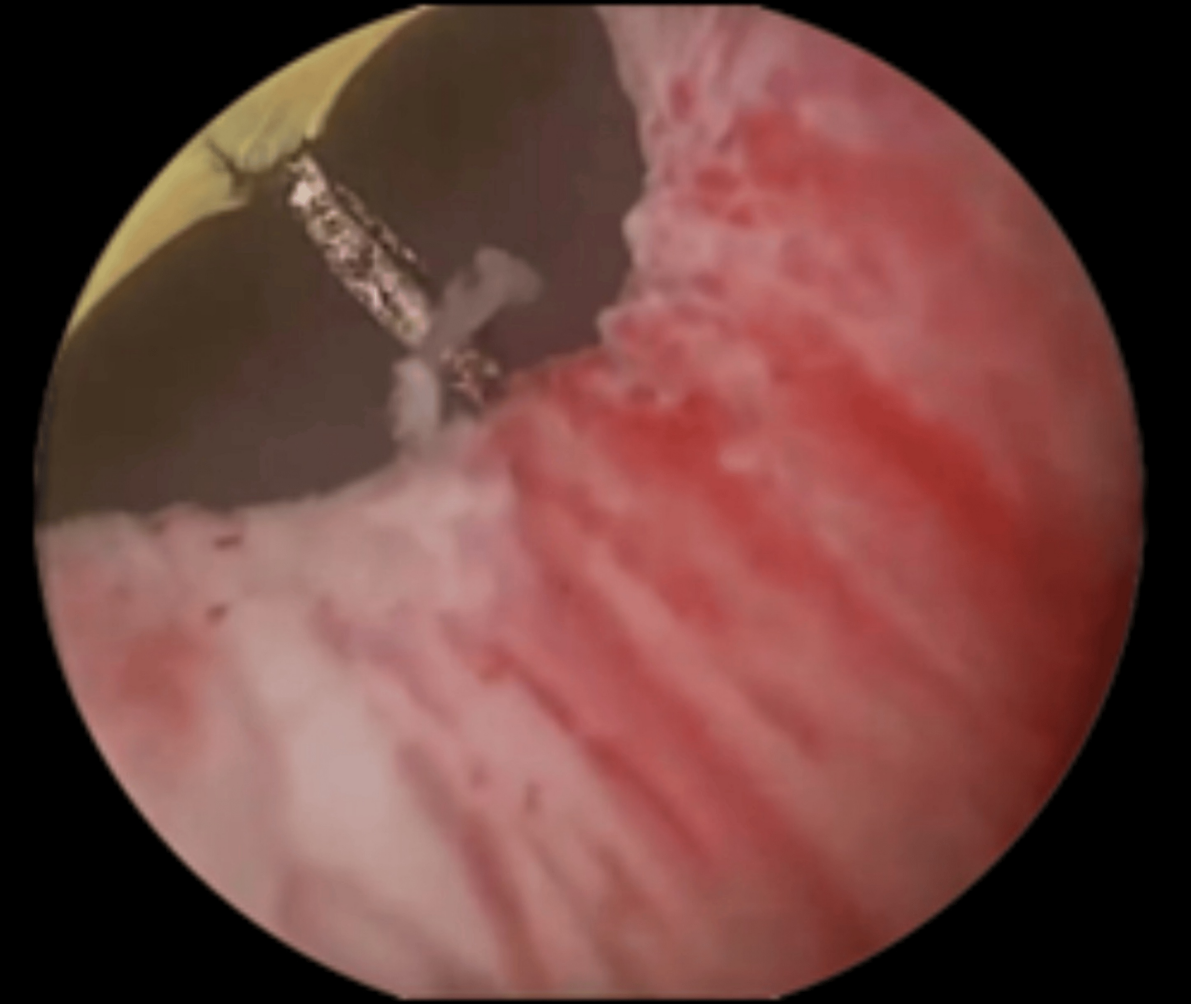
Vertical diathermy electrode (Collin’s knife) at the 5 o’clock position at the bladder neck. The initial step of the surgery involves marking the incision, proximally, 1 cm distal to the ureteric orifices on each side, and distally, up to the proximal third of the urethra. This ensures safety by preventing extension of the incision beyond the marked boundaries.

**Figure 3:**
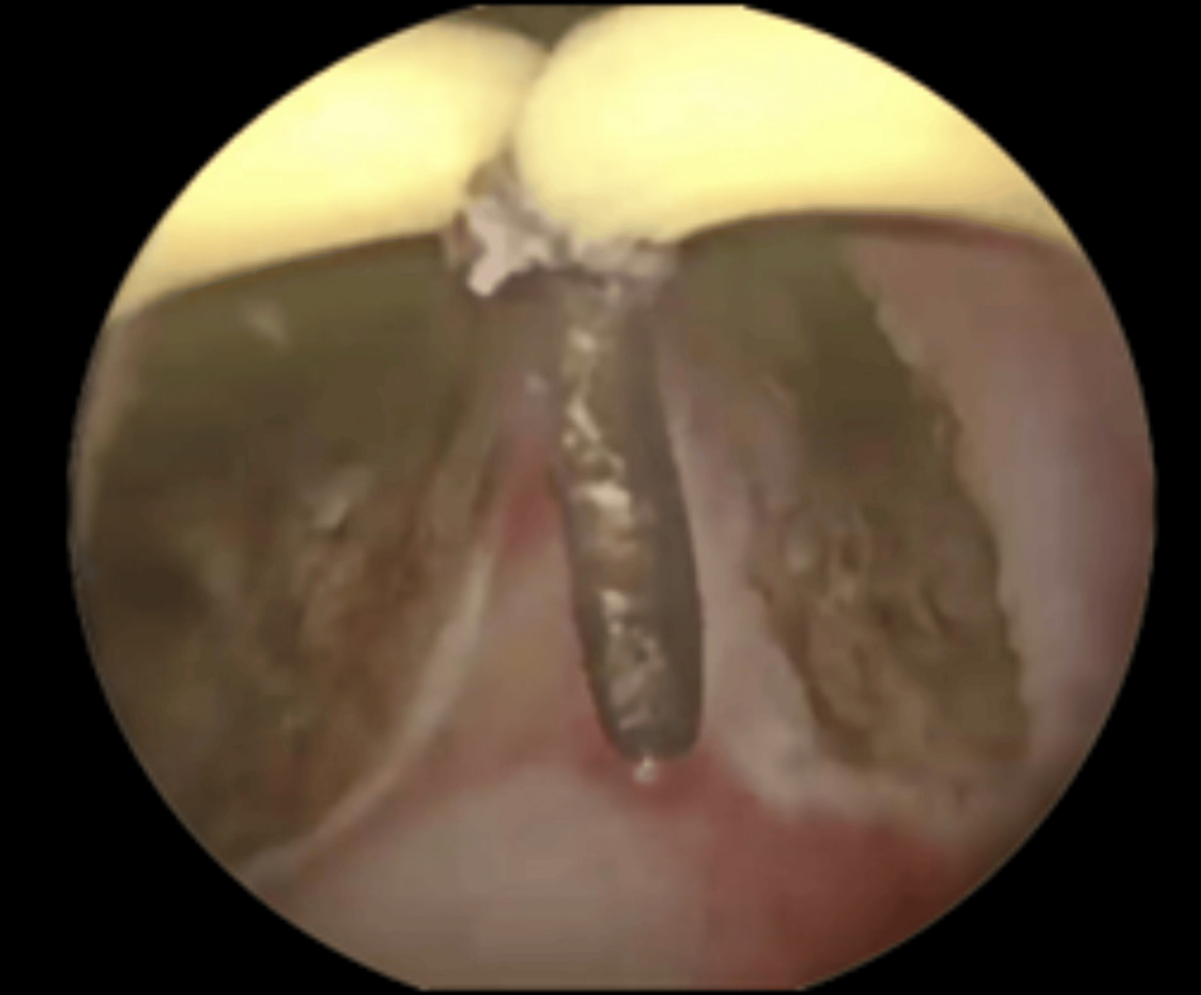
Incisions made at the 5 o’clock and 7 o’clock positions at the bladder neck. The Collin’s knife is moved from proximal to distal in repeated strokes, sequentially deepening the incision in a controlled fashion.

The operation (BNI) was performed in the standard lithotomy position. We began by marking the limits of the incision, proximally, 1 cm distal to the ureteric orifices on each side, and distally up to the proximal third of the urethra. Endoscopic incision was performed with a vertical diathermy electrode (Collin's knife) at the bladder neck and proximal urethra at the 5 o’clock and 7 o’clock positions (Figures 2-3). The incision was made deep enough to sufficiently divide the circular fibers, but not extending into the fat. An 18 Fr Foley catheter was inserted at the end of the procedure and removed on post-operative day 3.

Patients were followed up regularly, and data collection was done at 6 weeks and 6 months postoperatively. This included post-void residual volume (PVR) measurement, uroflowmetry, and the International Prostate Symptom Score (IPSS). Repeated multichannel urodynamic measurements were performed only in patients with unsatisfactory results. Success of BNI was defined as: an improvement in Qmax of ≥ 2 mL/sec, a reduction in PVR by ≥ 50%, a reduction in IPSS by ≥ 7, and an improvement in the Quality of Life (QoL) score.

Statistical analysis

Analysis of the tabulated data was done using Epi Info 7.0 software. Demographic variables were presented as means and SDs. Comparisons between Qmax, PVR, and IPSS pre- and post-operatively were done using ANOVA. Subsequently, univariate analysis was done to correlate various demographic parameters with the above-mentioned dependent variables. Differences of P < 0.05 were considered statistically significant.

## Results

Within the five-year study period, transurethral BNI was performed in 20 female patients who were diagnosed with PBNO. Follow-up data at six weeks and six months post-operatively were available for all 20 patients included in the study.

The mean age of the patients who underwent BNI was 50.58 ± 10.79 years. The mean BMI was 24.75 ± 2.38 kg/m². Fourteen (70%) patients had a history of UTI. Seven patients (35%) had co-morbidities, of which five (25%) had hypertension and two (10%) had type 2 diabetes mellitus. Seven patients (35%) had a previous history of surgery: five (25%) had undergone a lower segment caesarean section (LSCS), one (5%) had undergone percutaneous nephrolithotomy (PCNL), and one (5%) had undergone laparoscopic cholecystectomy. Four patients (20%) had a history of constipation. Six patients (30%) had previously taken a trial of alpha-blocker therapy for their symptoms (Table 1).

**Table 1:** Demographics and peri-operative data. Data are presented as Mean ± SD. Qmax: Maximum urinary flow rate; PVR: Post-void residual urine volume; IPSS: International Prostate Symptom Score.

Variables	Value
Number of cases	20
Age (years)	50.95 ± 10.63
Duration of symptoms (weeks)	19 ± 2.78
Comorbidities (N, %)	7 (35%)
History of UTI (N, %)	14 (70%)
Past surgical history (N, %)	7 (35%)
BMI (kg/m^2^)	24.75 ± 2.38
Serum creatinine (mg/dL)	0.83 ± 0.33
Qmax (mL/sec)	6.49 ± 2.63
PVR (mL)	202.11 ± 70.20
IPSS	26.95 ± 2.84
Mean operative time (minutes)	15 ± 2.8

At six months post-surgery, the mean Qmax increased from 6.49 ± 2.63 mL/sec to 12.41 ± 2.42 mL/sec (p-value: 0.0421; Table 2). The mean PVR decreased from 202.11 ± 70.20 mL to 53.11 ± 14.78 mL (p-value: 0.0152; Table 2). The mean IPSS decreased from 26.95 ± 2.84 to 14.74 ± 3.23 (p-value: 0.0325; Table 2). The mean QoL score improved from 4.70 ± 0.80 to 1.60 ± 0.12 (p-value: 0.0067; Table 2). Univariate analysis (Table 3) revealed no significant association between fixed parameters (serum hemoglobin, serum creatinine, duration of symptoms, history of UTI) and outcome measures (Qmax, PVR, IPSS).

**Table 2:** Comparison of parameters (pre-operative vs. six months post-surgery). Data are presented as mean ± SD. ANOVA was applied for statistical analysis. *p < 0.05 is considered statistically significant. The F-values indicate the degree of variance between the pre-operative and post-operative (six months) groups for each variable. A higher F-value suggests a greater difference between the groups relative to the variance within the groups. Qmax: Maximum urinary flow rate; PVR: Post-void residual urine volume; IPSS: International Prostate Symptom Score; QoL: Quality of Life.

Variable	Pre-operative	Post-operative (6 months)	F-value	P-value
Qmax (mL/sec)	6.49 ± 2.63	12.41 ± 2.42	54.87	0.0421*
PVR (mL)	202.11 ± 70.20	53.11 ± 14.78	82.84	0.0152*
IPSS	26.95 ± 2.84	14.74 ± 3.23	161.19	0.0325*
QoL	4.70 ± 0.80	1.60 ± 0.12	293.70	0.0067*

**Table 3:** Univariate analysis of Qmax, PVR, and IPSS at 6 months with various parameters to determine p-value. Univariate logistic regression was applied to analyze the association between fixed parameters and outcome measures. P < 0.05 is considered statistically significant. No statistically significant associations were observed. Qmax: Maximum urinary flow rate; PVR: Post-void residual urine volume; IPSS: International Prostate Symptom Score.

Parameter	Qmax (6 months)	PVR (6 months)	IPSS (6 months)
Serum Hemoglobin	0.0948	-0.0071	-0.0071
Duration of Symptoms	-0.0015	0.0873	0.0873
Serum Creatinine	-0.1509	-0.1618	-0.1618
History of UTI	0.323	0.2149	0.2149

One patient developed post-operative urinary retention upon removal of the Foley catheter on post-operative day 3. She underwent a repeat urodynamic study (UDS) and a re-do BNI (Clavien-Dindo grade IIIa complication). There were no other procedure-related complications. Fourteen (70%) patients met the pre-defined success parameter for increase in Qmax. Eighteen (90%) patients met the pre-defined success parameter for reduction in PVR. Seventeen (85%) patients had an improvement in IPSS of >7. Eighteen (90%) patients had an improvement in the QoL score.

## Discussion

Compared to males, PBNO is uncommon in females. Video urodynamics (VDUS) is at the forefront of diagnosing functional bladder disorders like PBNO. However, in many centers where VDUS is unavailable, conventional UDS along with cystoscopy and micturating cystourethroscopy offers an adequate armamentarium for diagnosis.

BNI for PBNO was first described by Turner-Warwick R et al. in 1971 [10]. Various methods have since been described. A conventional approach is to make the incisions at the 5 o’clock and 7 o’clock positions. The length of the incision is limited to the proximal urethra; the mid-urethra is spared, as it is considered the zone of maximum continence. The depth of incision varies, but in our study, the depth was limited to the circular muscle fibers. We refrained from increasing the depth beyond the circular fibers and into the fat to avoid the risk of VVF. Blaivas JG et al. believed that it is important to remember that a failure to relieve obstruction is generally correctable by repeat resections, whereas the incontinence that might result from overzealous therapy requires more extensive treatment [11]. Jin XB et al. performed transurethral incisions of the bladder neck with modification of the incisions at four different sites on the bladder neck (the 3, 6, 9, and 12 o’clock positions), and patients underwent regular urethral dilations post-operatively. They suggested that a modified BNI is effective in the long term in relieving voiding LUTS without urinary incontinence [12]. In a study by Zhang P et al., 84 patients diagnosed with PBNO on VDUS were enrolled for BNI and followed up for 1 year post-surgery. Sixty-three patients underwent incisions at the 5 o’clock and 7 o’clock positions, of which 3 patients (4.7%) developed VVF. Twenty-one patients underwent incisions at the 2 o’clock and 10 o’clock positions, and none of those patients developed VVF [13]. In our study, incisions were made at the 5 o’clock and 7 o’clock positions; however, none of the patients developed VVF (Table 4). In the study by Zhang P et al., 6 (7.14%) patients underwent multiple BNIs due to recurrent bladder neck obstruction, and 4 (4.7%) patients experienced stress urinary incontinence (SUI) [13]. In our study, 1 (5%) patient developed recurrent bladder neck obstruction and underwent re-do BNI (Table 4). None of the patients in our study developed SUI (Table 4). We attribute the low rate of complications in our study to the precise technique of performing BNI as described.

**Table 4:** Post-operative complications associated with bladder neck incision. Sample size, N = 20.

Complication	Frequency (%)
Urinary incontinence	Nil
Vesicovaginal fistula	Nil
Need for re-surgery	1 (5%)

In our study, we noticed a slight deterioration in the objective (Qmax and PVR) and subjective (IPSS and QoL score) parameters at 6 months post-surgery compared to 6 weeks post-surgery. However, this difference was not statistically significant (Table 5). One patient in our study who underwent BNI had an underactive bladder on UDS. There was non-resolution of symptoms as well as minimal change in flow rate and PVR after BNI.

**Table 5:** Comparison of parameters (six weeks post-surgery vs. six months post-surgery). Data are presented as mean ± SD. ANOVA was applied for statistical analysis. P < 0.05 is considered statistically significant. The F-values indicate the degree of variance between the two post-operative time points (6 weeks and 6 months) for each variable. Lower F-values suggest less variance between the groups. BNI: Bladder neck incision; Qmax: Maximum urinary flow rate; PVR: Post-void residual urine volume; IPSS: International Prostate Symptom Score; QoL: Quality of Life.

Variable	6 Weeks Post-BNI	6 Months Post-BNI	F-value	P-value
Qmax (mL/sec)	14.54 ± 3.68	12.41 ± 2.42	4.68	0.268
PVR (mL)	52.70 ± 14.96	56.11 ± 14.78	0.53	0.343
IPSS	12.80 ± 3.24	14.74 ± 3.23	3.6	0.649
QoL	1.57 ± 0.21	1.60 ± 0.12	0.31	0.237

Limitations

This was a single-center study with a small sample size of twenty patients, which limits the generalizability of the findings. The follow-up period was limited to 6 months, which restricts reporting on long-term complications.

## Conclusions

BNI for PBNO in females provides satisfactory results. A precise technique of BNI, including careful consideration of the depth and distal extent of the incision, helps avoid complications such as vesicovaginal fistula and urinary incontinence. Recurrent bladder neck obstruction is the most common complication reported in contemporary studies and is effectively managed with repeat BNI. The decision to perform BNI in patients with underactive bladder should be individualized.

A multicentric study with a larger sample size is recommended to generalize the findings of our study to a broader population and to enhance statistical precision.

## References

[1] Kuo HC (2004). Urodynamic parameters for the diagnosis of bladder outlet obstruction in women. Urol Int.

[2] Groutz A, Blaivas JG, Chaikin DC (2000). Bladder outlet obstruction in women: definition and characteristics. Neurourol Urodyn.

[3] Massey JA, Abrams PH (1988). Obstructed voiding in the female. Br J Urol.

[4] Goldman HB, Zimmern PE (2006). The treatment of female bladder outlet obstruction. BJU Int.

[5] Yande S, Joshi M (2011). Bladder outlet obstruction in women. J Midlife Health.

[6] Brucker BM, Fong E, Shah S, Kelly C, Rosenblum N, Nitti VW (2012). Urodynamic differences between dysfunctional voiding and primary bladder neck obstruction in women. Urology.

[7] Nitti VW (2005). Primary bladder neck obstruction in men and women. Rev Urol.

[8] Abrams P, Cardozo L, Fall M (2002). The standardisation of terminology of lower urinary tract function: report from the Standardisation Sub-committee of the International Continence Society. Neurourol Urodyn.

[9] Axelrod SL, Blaivas JG (1987). Bladder neck obstruction in women. J Urol.

[10] Turner-Warwick R, Whiteside CG, Worth PH, Milroy EJ, Bates CP (1973). A urodynamic view of the clinical problems associated with bladder neck dysfunction and its treatment by endoscopic incision and trans-trigonal posterior prostatectomy. Br J Urol.

[11] Blaivas JG, Flisser A, Tash JA (2004). Treatment of primary bladder neck obstruction in women with transurethral resection of the bladder neck. J Urol.

[12] Jin XB, Qu HW, Liu H, Li B, Wang J, Zhang YD (2012). Modified transurethral incision for primary bladder neck obstruction in women: a method to improve voiding function without urinary incontinence. Urology.

[13] Zhang P, Wu ZJ, Xu L, Yang Y, Zhang N, Zhang XD (2014). Bladder neck incision for female bladder neck obstruction: long-term outcomes. Urology.

